# Polyphenol Intake and Epithelial Ovarian Cancer Risk in the European Prospective Investigation into Cancer and Nutrition (EPIC) Study

**DOI:** 10.3390/antiox10081249

**Published:** 2021-08-04

**Authors:** Catalina Londoño, Valerie Cayssials, Izar de Villasante, Marta Crous-Bou, Augustin Scalbert, Elisabete Weiderpass, Antonio Agudo, Anne Tjønneland, Anja Olsen, Kim Overvad, Verena Katzke, Matthias Schulze, Domenico Palli, Vittorio Krogh, Maria Santucci de Magistris, Rosario Tumino, Fulvio Ricceri, Inger T. Gram, Charlotta Rylander, Guri Skeie, Maria-Jose Sánchez, Pilar Amiano, José María Huerta, Aurelio Barricarte, Hanna Sartor, Emily Sonestedt, Anders Esberg, Annika Idahl, Yahya Mahamat-Saleh, Nasser Laouali, Marina Kvaskoff, Renée Turzanski-Fortner, Raul Zamora-Ros

**Affiliations:** 1 Unit of Nutrition and Cancer, Cancer Epidemiology Research Programme, Catalan Institute of Oncology, Bellvitge Biomedical Research Institute (IDIBELL), 08908 Barcelona, Spain; catalondonocanola@gmail.com (C.L.); valerie.cayssials@gmail.com (V.C.); idevillasante@idibell.cat (I.d.V.); marta.crous@iconcologia.net (M.C.-B.); a.agudo@iconcologia.net (A.A.); 2 Department of Public Health, Faculty of Veterinary, University of the Republic, Montevideo 11600, Uruguay; 3 Department of Quantitative Methods, Faculty of Medicine, University of the Republic, Montevideo 11600, Uruguay; 4 International Agency for Research on Cancer (IARC), 69372 Lyon, France; ScalbertA@iarc.fr (A.S.); WeiderpassE@iarc.fr (E.W.); 5 Unit of Diet, Genes and Environment, Danish Cancer Society Research Center, 2100 Copenhagen, Denmark; annet@cancer.dk (A.T.); anja@cancer.dk (A.O.); 6 Department of Public Health, Section for Epidemiology, Aarhus University, 8000 Aarhus, Denmark; ko@ph.au.dk; 7 Division of Cancer Epidemiology, German Cancer Research Center (DKFZ), 69120 Heidelberg, Germany; V.Katzke@Dkfz-Heidelberg.de (V.K.); r.fortner@dkfz-heidelberg.de (R.T.-F.); 8 Department of Molecular Epidemiology, German Institute of Human Nutrition Potsdam-Rehbruecke, 14558 Nuthetal, Germany; mschulze@dife.de; 9 Institute of Nutritional Science, University of Potsdam, 14469 Potsdam, Germany; 10 Cancer Risk Factors and Life-Style Epidemiology Unit, Institute for Cancer Research, Prevention and Clinical Network-ISPRO, 50139 Florence, Italy; d.palli@ispro.toscana.it; 11 Epidemiology and Prevention Unit, Fondazione IRCCS Istituto Nazionale dei Tumori, 20133 Milan, Italy; Vittorio.Krogh@istitutotumori.mi.it; 12 Department of Clinical and Experimental Medicine, Federico II University, 80138 Naples, Italy; masantuc@unina.it; 13 Cancer Registry and Histopathology Unit, “Civic M.P. Arezzo” Hospital ASP, 97100 Ragusa, Italy; rtuminomail@gmail.com; 14 Department of Clinical and Biological Sciences, University of Turin, 10124 Turin, Italy; fulvio.ricceri@unito.it; 15 Unit of Epidemiology, Regional Health Service ASL TO3, 10095 Grugliasco, Italy; 16 Department of Community Medicine, Faculty of Health Sciences, University of Tromsø, The Arctic University of Norway, 9019 Tromsø, Norway; inger.gram@uit.no (I.T.G.); charlotta.rylander@uit.no (C.R.); guri.skeie@uit.no (G.S.); 17 Escuela Andaluza de Salud Pública (EASP), 18011 Granada, Spain; mariajose.sanchez.easp@juntadeandalucia.es; 18 Instituto de Investigación Biosanitaria ibs.GRANADA, 18012 Granada, Spain; 19 Centro de Investigación Biomédica en Red de Epidemiología y Salud Pública (CIBERESP), 28029 Madrid, Spain; epicss-san@euskadi.eus (P.A.); jmhuerta.carm@gmail.com (J.M.H.); aurelio.barricarte.gurrea@navarra.es (A.B.); 20 Department of Preventive Medicine and Public Health, University of Granada, 18011 Granada, Spain; 21 Ministry of Health of the Basque Government, Sub-Directorate for Public Health and Addictions of Gipuzkoa, 20013 San Sebastian, Spain; 22 Public Health Division of Gipuzkoa, BioDonostia Research Institute, 20014 San Sebastian, Spain; 23 Department of Epidemiology, Murcia Regional Health Council, IMIB-Arrixaca, 30008 Murcia, Spain; 24 Navarra Public Health Institute, 31003 Pamplona, Spain; 25 Navarra Institute for Health Research (IdiSNA), 31008 Pamplona, Spain; 26 Diagnostic Radiology Unit, Lund University, 20502 Malmö, Sweden; hanna.sartor@med.lu.se; 27 Department of Medical Imaging and Physiology, Skåne University Hospital, 21428 Malmö, Sweden; 28 Nutritional Epidemiology, Department of Clinical Sciences Malmö, Lund University, 21428 Malmö, Sweden; emily.sonestedt@med.lu.se; 29 Department of Odontology, Umeå University, 90187 Umeå, Sweden; anders.esberg@umu.se; 30 Department of Clinical Sciences, Obstetrics and Gynecology, Umeå University, 90187 Umeå, Sweden; annika.idahl@umu.se; 31 Institut Gustave Roussy, 94805 Villejuif, France; yahya.mahamat-saleh@gustaveroussy.fr (Y.M.-S.); nasser.laouali@gustaveroussy.fr (N.L.); marina.kvaskoff@gustaveroussy.fr (M.K.); 32 Exposome and Heredity Team, CESP, Paris-Saclay University, UVSQ, INSERM, 94805 Villejuif, France

**Keywords:** ovarian cancer, polyphenols, flavonoids, intake, cohort, EPIC

## Abstract

Despite some epidemiological evidence on the protective effects of polyphenol intake on epithelial ovarian cancer (EOC) risk from case-control studies, the evidence is scarce from prospective studies and non-existent for several polyphenol classes. Therefore, we aimed to investigate the associations between the intake of total, classes and subclasses of polyphenols and EOC risk in a large prospective study. The study was conducted in the European Prospective Investigation into Cancer and Nutrition (EPIC) cohort, which included 309,129 adult women recruited mostly from the general population. Polyphenol intake was assessed through validated country-specific dietary questionnaires and the Phenol-Explorer database. During a mean follow-up of 14 years, 1469 first incident EOC cases (including 806 serous, 129 endometrioid, 102 mucinous, and 67 clear cell tumours) were identified. In multivariable-adjusted Cox regression models, the hazard ratio in the highest quartile of total polyphenol intake compared with the lowest quartile (HR_Q4vsQ1_) was 1.14 (95% CI 0.94–1.39; *p*-trend = 0.11). Similarly, the intake of most classes and subclasses of polyphenols were not related to either overall EOC risk or any EOC subtype. A borderline statistically significant positive association was observed between phenolic acid intake (HR_Q4vsQ1_ = 1.20, 95% CI 1.01–1.43; *p*-trend = 0.02) and EOC risk, especially for the serous subtype and in women with obesity, although these associations did not exceed the Bonferroni correction threshold. The current results do not support any association between polyphenol intake and EOC in our large European prospective study. Results regarding phenolic acid intake need further investigation

## 1. Introduction

Ovarian cancer is the seventh most common cancer worldwide in women, and it is the most lethal gynaecological malignancy [1]. Epithelial ovarian cancer (EOC) represents 90% of all ovarian cancers. The five-year survival rate of EOC is low (approximately 40% in Europe) [2], and there are currently no strategies for early detection. Consequently, most patients are diagnosed with advanced-stage disease. Furthermore, there are relatively few protective/risk factors that have been established for EOC, especially hormonal and reproductive factors (e.g., parity and oral contraceptive use) [3], although recent studies have reported different risk factor profiles (e.g., body fatness [4] and smoking [5]) by tumour histotype [3]. Dietary factors, such as a plant-based diet, have been suggested to play a role in EOC aetiology, although until now epidemiological evidence is still limited and mainly inconclusive [6].

Polyphenols are bioactive compounds abundant in some plant-based foods, such as tea, coffee, wine, fruit, vegetables, whole-grain cereals, and cocoa [7]. They are chemically divided into 4 main classes: flavonoids, phenolic acids, lignans, and stilbenes. The mean intake of polyphenols in Europe ranges from 584 mg/day in Greek women to 1786 mg/day in Danish men [8]. These abundant compounds may have chemopreventive effects, through their weak anti-estrogenic and estrogenic-mimetic effects [9] and the modulation of enzyme activities and signal transduction pathways related to cellular proliferation, differentiation, apoptosis, inflammation, angiogenesis, and metastasis [10,11].

In a recent meta-analysis, higher intakes of total flavonoids were associated with a lower ovarian cancer risk [12]. Intakes of isoflavones, and flavonols were also related with lower risk of ovarian cancer [12], but these findings were based mainly on case-control studies. In contrast, no association was observed between phytoestrogens (including lignans, isoflavones and coumestrol) intake and ovarian cancer risk in a Swedish cohort [13]. To our knowledge, no studies have investigated the relationships between other polyphenol classes (e.g., phenolic acids, stilbenes, and other minor subclasses) and EOC risk.

In the current study, we aimed to examine associations between the intake of total polyphenols and individual polyphenol subclasses and EOC risk and by histological subtype in the European Prospective Investigation into Cancer and Nutrition (EPIC) study, a large cohort with a high variability in polyphenol intake and around two decades of follow-up [8].

## 2. Materials and Methods

### 2.1. Study Population

EPIC is an on-going multi-centre cohort study aimed to evaluate the associations between dietary, lifestyle and genetic factors and cancer risk [14]. The study enrolled participants between 1992 and 2000, mostly aged between 35–70 years, from 23 centres in 10 European countries (Denmark, France, Germany, Greece, Italy, the Netherlands, Norway, Spain, Sweden, and United Kingdom) (Supplementary Materials, Figure S1). The participants were mostly chosen from the general population, except for France (women who were health insurance members), Utrecht and Florence (women attending breast cancer screening), Oxford (mostly health-conscious volunteers including a large proportion of vegetarians), and some centres in Spain and Italy where the participants were mostly blood donors.

The EPIC study recruited 521,324 participants, of which 367,898 were women. Exclusions before the beginning of the analyses included: participants with missing or null follow-up time or having a prevalent cancer other than non-melanoma skin cancer at baseline (*n* = 23,913); had a bilateral oophorectomy (*n* = 10,761); participants with incomplete information on diet and lifestyle (*n* = 3137); and participants with dietary data considered to be implausible (i.e., participants in the highest and lowest 1% of the distribution for the ratio between energy intake to estimated energy requirement, *n* = 6186); participants from Greece, (data not provided for the current study, *n* = 14,772). Therefore, our analysis included 309,129 women.

### 2.2. Follow-Up and Case Ascertainment

Incident cancer cases were identified through population cancer registries in all countries except in all centres in France, Germany, and Naples (Italy) where cases were identified through active follow-up, directly from the participants or next of kin, and confirmed by a combination of methods, such as health insurance records, and cancer and pathology registries. Vital status was obtained from mortality registries at the regional or national level. The 10th Revision of the International Statistical Classification of Diseases, Injuries, and Causes of Death (ICD-10) was used to code ovarian (code C56.9), fallopian tube (code C57.0), and peritoneal cancers (code C48). Histologically, EOCs were classified as serous, not otherwise specified (including adenocarcinomas, carcinomas, cystadenocarcinoma, and others), endometrioid, mucinous, and clear cell (*n* = 67, 4.6%).

### 2.3. Dietary and Lifestyle Assessment

At baseline, habitual dietary information regarding the previous year was collected using country/centre specific dietary questionnaires [14]. According to the centres, quantitative or semi-quantitative methods were applied. In Malmö (Sweden), a combination of a 7-day record and a semi-quantitative food frequency questionnaire was administered. Questionnaires were mainly self-reported except for all centres in Spain, and Naples and Ragusa (Italy) where they were administered by trained interviewers. The relative validity and reproducibility of these questionnaires was previously demonstrated for food groups, macro- and micronutrients, but not for polyphenols [15]. Daily food intakes were calculated in g/day (g/d). Nutrients (g/d) and total energy (kcal/d) intakes were computed using the standardised EPIC Nutrient Database [16].

Dietary polyphenol intake was estimated using the Phenol-Explorer database [17], taking into account cooking and processing of foods via retention factors [18], as previously described [19]. The content of polyphenols was expressed in mg/100 g of food fresh weight and expressed as they are found in nature (mainly glycosides and esters). Total polyphenols were calculated as the sum of all classes of polyphenols: flavonoids [anthocyanidins, chalcones, dihydrochalcones, dihydroflavonols, flavanols (including flavan-3-ol monomers, proanthocyanidins, theaflavins), flavanones, flavones, flavonols, and isoflavones], phenolic acids (hydroxybenzoic acids, hydroxycinnamic acids, and hydroxyphenylacetic acids), lignans, stilbenes, and other minor polyphenols (alkylphenols, tyrosols, alkymethoxyphenols, furanocoumarins, hydroxybenzaldehydes, and hydroxycoumarins).

Lifestyle questionnaires were used to obtain information on education, smoking status and intensity, alcohol consumption, physical activity levels that were coded using the Cambridge Physical Activity Index [20], and reproductive variables. Anthropometry (weight and height) was measured at recruitment by trained personnel, with the exception of Oxford (United Kingdom), Norway, and France, where data was self-reported [14].

### 2.4. Statistical Analysis

Polyphenol intake was analysed as categorical variables based on quartiles of the distribution among the entire EPIC cohort. Tests for linear trend were performed by assigning medians of each quartile as scores. Polyphenol intake was also analysed as continuous variables, after log_2_ transformation to reduce skewness of intake distributions. One unit corresponded to a doubling in intake.

Cox proportional hazard models were used to estimate hazard ratios (HR) and 95% confidence intervals (CI) between total, classes and subclasses of polyphenol intakes and EOC risk. Age was the primary time variable in all models. Entry time was age at recruitment and exit time was age at diagnosis of EOC, death, or censoring date (loss or end of follow-up), whichever occurred first. Tests and graphs based on Schoenfeld residuals were used to assess proportional hazards assumptions, which were satisfied. The basic model (model 1) was stratified by age at recruitment (1-year interval) and study centre to control for differences in questionnaires and follow-up procedures. The multivariable model (model 2) was additionally adjusted for covariates that were selected a priori: body mass index (BMI, kg/m^2^), smoking status (never, former, current smoker, and not specified), alcohol consumption (g/d), education level (none, primary school, technical/professional school, secondary school, university or higher, and not specified), physical activity (inactive, moderately inactive, moderately active, active, and not specified), menopausal status and type (pre-, peri-, and post-menopausal), age at menopause (≤45, ≥46–≤50, >50 years, and not specified), age at first menstrual period (≤11, 12, 13, 14, ≥15 years, and not specified), use of oral contraceptives (yes, no and unknown), duration of oral contraceptives (≤1, 2–4, 5–7, 8–10, ≥10 years, and not specified), hormone replacement therapy use (HRT; yes, no and unknown), and full-term pregnancies (0, 1–2, 3–4, >4, and not specified). Additionally, Cox models were further adjusted for total energy (kcal/d) intake or polyphenol intake was evaluated as nutrient density (mg/2000 kcal d) [21]. As we believe crude values of polyphenol intake are more adequate than energy-adjusted values and the results were almost identical, we only presented the results of the models without the co-variable total energy intake.

Separate exploratory analyses were performed for invasive EOC histological subtypes: serous, endometrioid, mucinous and clear cell tumours. Possible interactions of intake of total polyphenol, flavonoids and phenolic acids with BMI (<25, ≥25–29.9, and ≥30 kg/m^2^), tobacco smoking status (never, former, and current smokers), physical activity (inactive, moderately inactive, moderately active, and active), education level (none, primary school, technical/professional school, secondary school, and university or higher), and menopausal status (pre-, peri-, and post-menopausal) were tested by including an interaction term in the multi-adjusted models. Separate BMI-specific models were fitted because a significant interaction between BMI categories and polyphenol intake was detected. Further separate analysis was conducted between coffee consumer and non-consumers because coffee is the main contributor to phenolic acids. Sensitivity analyses were performed by repeating the models after the exclusion of EOC cases diagnosed during the first 2 years of follow-up, since participants may have changed their diets in the pre-diagnostic period. For all analyses, *p*-values < 0.05 were considered as statistically significant. To account for multiple testing for the subclasses of polyphenols, Bonferroni correction was used and then results were considered statistically significant if *p*-value < 0.002 (i.e., <0.05/23, the number of tests for the intakes of all polyphenol subclasses). Statistical analyses were conducted by using STATA, version 13.0, software (StataCorp, College Station, TX, USA).

## 3. Results

Overall, 1469 out of 309,129 women were diagnosed with a first primary incident EOC during the 14-years of mean follow-up (Table 1), of which 806 were serous (54.9%), 365 not otherwise specified (24.8%), 129 endometrioid (8.8%), 102 mucinous (6.9%), and 67 clear cell EOC (4.6%). The median (10th and 90th percentile) of total polyphenol intake was 1070 (751–1476) mg/d. Large variability in the intake of polyphenols was observed among women from the nine participating European countries. Phenolic acids were the main contributors to total polyphenols (50.5%), followed by flavonoids (45.4%), other minor polyphenol classes (3.8%), lignans (0.16%) and stilbenes (0.14%). Women in the highest quartile of polyphenol intake were older, more physically active, had a lower BMI, higher educational level, and had a lower proportion of never smokers, were more likely to be post-menopausal and users of HRT and oral contraceptives than those with lower total polyphenol intakes (Supplementary Materials, Table S1).

No statistically significant association was observed between the extreme quartiles of the intake of total polyphenols (HR_Q4vsQ1_ = 1.14, 95% CI 0.94–1.39; *p*-trend = 0.11) and flavonoids (HR_Q4vsQ1_ = 1.06, 95% CI 0.88–1.29; *p*-trend = 0.50) and the risk of EOC. However, a positive association was detected with phenolic acid intake (HR_Q4vsQ1_ = 1.20, 95% CI 1.01–1.43; *p*-trend = 0.02), but it did not reach the Bonferroni threshold (*p* = 0.002) (Table 2). Identical results for total polyphenols and phenolic acids were observed additionally adjusting for total energy intake. Similar results were also found using nutrient density (mg/2000 kcal d) models, including for phenolic acids (HR_Q4vsQ1_ = 1.25, 95% CI 1.06–1.49; *p*-trend = 0.005). No statistically significant results were observed after excluding 173 cases who were diagnosed with EOC within the first 2 years of follow-up for total polyphenols (HR_Q4vsQ1_ = 1.14, 95% CI 0.94–1.39; *p*-trend = 0.10), flavonoids (HR_Q4vsQ1_ = 1.15, 95% CI 0.93–1.41; *p*-trend = 0.19) and phenolic acids (HR_Q4vsQ1_ = 1.13, 95% CI 0.95–1.36; *p*-trend = 0.11). No associations were observed in the exploratory analyses between the intake of total polyphenols, flavonoids and phenolic acids and the subtypes of EOC, including serous, endometrioid, mucinous, and clear cell tumours (Table 3), although results were borderline significant for phenolic acid intake and serous EOC risk (HR_Q4vsQ1_ = 1.24, 95% CI 0.99–1.56; *p*-trend = 0.04).

No statistically significant relationships were found between any of the intake of 23 polyphenol subclasses and EOC risk (Table 2), except for a borderline statistically significant association with the hydroxycinnamic acid intake (HR_Q4vsQ1_ = 1.16, 95% CI 0.99–1.37; *p*-trend = 0.04), the main contributor to phenolic acids (92.5%). After dividing our results into coffee consumers and non-consumers, stronger non-significant positive associations between main polyphenol classes and EOC risk were observed compared to the results in the entire population (Supplementary Materials, Table S2). Conversely, non-significant inverse associations with the main classes of polyphenols and EOC risk were detected in coffee non-consumers, but the number of EOC cases was low (*n* = 96).

The risk estimates for total polyphenol, flavonoid and phenolic acid intakes and EOC risk were not significantly modified by tobacco consumption, physical activity, educational level, and menopause status. Interactions between BMI categories and total polyphenol and phenolic acid intake (*p* for interaction = 0.018 and 0.091, respectively) in relation to EOC risk were noted. A stronger positive association between both total polyphenol and phenolic acid intake and EOC risk was observed across increasing BMI categories although a borderline significantly association was only observed with phenolic acid intake in women with BMI ≥ 30 (HR_Q4vsQ1_ = 1.57, 95% CI 0.99–2.51; *p*-trend = 0.02) (Supplementary Materials, Table S3)

## 4. Discussion

In this large prospective study, we observed no statistically significant associations between the intake of total polyphenol and either risk of overall EOC or EOC histological subtypes (e.g., serous, endometrioid, mucinous and clear cell tumours). To our knowledge, this is the first study evaluating such associations.

Phenolic acid intake, the main contributor to total polyphenols, was borderline non-statistically significant associated with both EOC risk and the serous subtype after applying the Bonferroni correction. Moreover, the statistical significance of this association disappeared after excluding the EOC cases diagnosed during the first 2 years of follow-up in the sensitivity analysis. Coffee consumption is, by far, the main food source of phenolic acids [22]. In coffee consumers, the results were slightly stronger than in the entire cohort, although results were still basically non-significant. In previous studies, such as the Prostate, Lung, Colorectal, and Ovarian cancer (PLCO) cohort [23], a Canadian population-based case control study [24] and a Mendelian randomisation study [25], no association between coffee intake and EOC risk was detected.

In the current study, the intake of total flavonoid and its subclasses were not related to EOC risk or its subtypes. Likewise, Cassidy et al. showed no association between total flavonoid intake and EOC risk in two prospective studies: the Nurses’ Health Study and the Nurses’ Health Study II [26]. In contrast to these findings, a recent meta-analysis found an 18% lower risk in women with highest intakes of flavonoids compared to those with lowest intakes [12], however, the authors mixed results from total and individual subclasses of flavonoids and from different study designs (cohorts and case-control studies). According to previous studies [12,26], flavonols is the flavonoid subclass with the strongest biological plausibility supporting chemopreventive effects against EOC risk; however, no association was observed in our study and in the prospective Women’s Health Study [27]. Isoflavone intake was inversely related to EOC risk in retrospective studies in Asian countries [12], where the intake of isoflavones is high (>30 mg/d) [28], but not in prospective studies in Western countries [13] where the intakes are very low (<2 mg/d) [29], as in our study.

No association was observed between lignan intake and EOC risk in our study. Similarly, null results were shown in a previous Swedish cohort [13] and in a US-based case-control study [30], whereas in another case-control study from the US a 57% lower ovarian cancer risk was observed between extreme quintiles of lignan intake [31]. Its potential underlying anticarcinogenic mechanism against EOC could be related to the weak estrogenic-mimetic effects [9], but lignans are consumed in very low amounts (<2 mg/d) [29], and therefore it is unlikely to have substantial effects at such low concentrations.

In the present study, no associations were observed between the intake of other minor subclasses of polyphenols (such as stilbenes, tyrosols, and alkylphenols) and EOC risk. Wine consumption, the most important food source of stilbenes [32], was not related to EOC risk in either the California Teachers Study [33] or in the Ovarian Cancer Association Consortium [34]. Olive oil consumption, the richest food source of tyrosols [8], was inversely associated with EOC risk in an Italian case-control study [35]. Similar protective results were found for whole grain cereals, the main food sources of alkylphenols [8], in a Polish case-control study [36]. Recall bias is a well-established drawback of case-control studies, especially to investigate dietary factors, and therefore further prospective studies are needed to evaluate the relationships with the intake of minor polyphenol subclasses.

We observed a potential effect modification in the association between total polyphenol intake and EOC risk by BMI in this study. A higher intake of polyphenols was more strongly associated with a higher EOC risk in women with BMI ≥ 30 than those with BMI < 30. Obesity (BMI ≥ 30), which is a modest risk factor of EOC, especially among never-users of hormone therapy or in endometroid carcinomas [3,4], is a low-grade inflammatory disease [37]. Thus, our initial hypothesis was that obese subjects may benefit more from the consumption of anti-inflammatory compounds, such as polyphenols, to counteract their greater grade of inflammation. However, our findings go against this hypothesis. Although these results did not exceed the Bonferroni threshold (*p*-value < 0.002), further research is needed to confirm or deny the potential positive associations between phenolic acid intake and EOC risk, particularly in women with obesity.

Major strengths of our study include its prospective design, the long follow-up, and large sample size and number of EOC cases, and the coverage of several European countries with large dietary heterogeneity. However, our study had some limitations. Firstly, our results may be influenced by measurement errors in the dietary assessment, which could have attenuated our findings; however, as we used validated country-specific dietary questionnaires [15], we consider this as unlikely. Moreover, the Phenol-Explorer is currently the most comprehensive food composition database on polyphenols available [17]. Polyphenol intake was potentially underestimated since supplements and drugs were not considered in the calculation, although their contribution is usually very low [38]. Secondly, diet was only assessed at baseline; thus, any potential dietary changes during follow-up are unaccounted for. In addition, some individuals may have modified their diet during the early prediagnostic period of the disease, but sensitivity analyses excluding incident cases diagnosed in the first 2 years of follow-up did not significantly alter the risk estimates. Finally, we have adjusted our models for several potential confounders, however, the presence of possible residual confounding cannot be excluded.

In conclusion, no association between total polyphenol intake and the risk of EOC, and its histological subtypes, was found in this large multi-centre European cohort. Null results were also observed with the intake of all classes and subclasses of polyphenols. For phenolic acid intakes, our study shows a borderline non-significant positive association with EOC risk, especially the serous subtype in women with obesity, but these sub-analysis findings should be interpreted with caution.

## Figures and Tables

**Table 1:** Number of total and histological subtypes of EOC cases and median (10th–90th percentile) of total polyphenol intake by country in the EPIC study.

Country	N	Overall EOC	Serous Tumours	Endometrioid Tumours	Mucinous Tumours	Clear Cell Tumours	NOS	Polyphenol Intake (mg/d)		
								Median	P10	P90
France	65,562	204	135	23	19	3	41	1320.7	692.0	2227.0
Italy	29,278	123	75	17	11	3	34	853.1	522.0	1291.1
Spain	23,504	86	52	10	5	8	19	673.2	324.0	1202.2
United Kingdom	50,877	303	131	19	24	20	127	1441.3	833.4	2035.9
Netherlands	26,116	128	75	11	8	4	40	1157.2	748.0	1604.5
Germany	26,542	85	55	7	9	0	16	1032.8	642.7	1645.6
Sweden	26,010	170	72	9	15	10	71	839.0	506.1	1294.0
Denmark	27,401	211	118	22	11	11	68	1556.0	956.4	1294.0
Norway	33,839	159	107	14	8	7	35	653.0	341.2	994.6
Total	309,129	1469	806	129	102	67	278	1070.5	532.1	1885.5

Abbreviations: EOC epithelial ovarian cancer; NOS not otherwise specified.

**Table 2:** Hazard ratios and 95% confidence intervals for epithelial ovarian cancer, according to quartile of intake of total, classes and subclasses of polyphenols in the EPIC study.

	Intake (mg/d)			Quartile 1	Quartile 2	Quartile 3	Quartile 4	*p*-Trend	Continuous (log_2_)
	Median	P10	P90	HR (95% CI)	HR (95% CI)	HR (95% CI)	HR (95% CI)		HR (95% CI)
Total polyphenols	1070.5	532.1	1885.5	1.00 (ref)	0.99 (0.84–1.16)	1.05 (0.88–1.26)	1.14 (0.94–1.39)	0.11	1.11 (1.00–1.23) *
Flavonoids	433.0	160.4	1039.8	1.00 (ref)	1.02 (0.87–1.19)	1.00 (0.84–1.20)	1.06 (0.88–1.29)	0.50	1.02 (0.95–1.09)
Flavanols	93.8	834.4	532.9	1.00 (ref)	1.00 (0.85–1.17)	0.98 (0.82–1.18)	1.04 (0.86–1.27)	0.55	1.00 (0.95–1.06)
Flavan-3-ol monomers	374.2	18.2	180.1	1.00 (ref)	0.98 (0.83–1.16)	0.97 (0.81–1.17)	1.03 (0.85–1.26)	0.50	1.00 (0.97–1.04)
Proanthocyanidins	443.3	128.7	313.7	1.00 (ref)	1.07 (0.91–1.25)	1.07 (0.90–1.26)	1.04 (0.86–1.26)	0.78	1.00 (0.93–1.06)
Theaflavins	88.7	0.0	36.9	1.00 (ref)	1.00 (0.80–1.25)	0.93 (0.77–1.12)	0.99 (0.81–1.21)	0.82	1.00 (0.97–1.02)
Flavonols	9.1	91.6	54.6	1.00 (ref)	1.04 (0.88–1.23)	0.98 (0.81–1.18)	1.04 (0.85–1.27)	0.69	0.99 (0.93–1.05)
Flavanones	3.8	88.8	55.9	1.00 (ref)	0.90 (0.77–1.04)	0.97 (0.84–1.12)	0.99 (0.85–1.15)	0.61	1.00 (0.97–1.02)
Anthocyanins	6.3	89.6	53.1	1.00 (ref)	1.03 (0.89–1.19)	1.05 (0.90–1.23)	1.01 (0.83–1.22)	1.00	1.02 (0.98–1.06)
Flavones	3.6	20.9	14.1	1.00 (ref)	0.95 (0.82–1.10)	1.04 (0.89–1.21)	1.02 (0.85–1.23)	0.57	1.02 (0.96–1.09)
Dihydrochalcones	0.3	4.7	6.4	1.00 (ref)	0.94 (0.80–1.10)	0.83 (0.71–0.97) *	1.00 (0.86–1.16)	0.80	0.99 (0.96–1.03)
Dihydroflavonols	0.0	6.0	10.2	1.00 (ref)	0.97 (0.83–1.13)	1.02 (0.87–1.20)	0.91 (0.73–1.13)	0.38	1.00 (0.98–1.01)
Isoflavonoids	0.0	1.9	0.1	1.00 (ref)	0.87 (0.75–1.01)	0.99 (0.84–1.17)	0.98 (0.79–1.21)	0.75	0.99 (0.97–1.01)
Phenolic acids	513.0	181.9	1067.4	1.00 (ref)	1.03 (0.88–1.21)	1.10 (0.94–1.29)	1.20 (1.01–1.43) *	0.02	1.07 (1.00–1.14) *
Hydroxycinnamic acids	131.5	1021.6	704.3	1.00 (ref)	1.02 (0.87–1.20)	1.13 (0.97–1.32)	1.16 (0.99–1.37)	0.04	1.05 (1.00–1.11) *
Hydroxybenzoics acids	2.1	133.4	66.8	1.00 (ref)	0.94 (0.79–1.12)	0.98 (0.80–1.21)	0.95 (0.77–1.18)	0.89	1.00 (0.97–1.04)
Hydroxyphenylacetic acids	0.0	0.4	0.2	1.00 (ref)	1.08 (0.94–1.25)	0.93 (0.79–1.08)	0.91 (0.73–1.14)	0.24	0.99 (0.96–1.01)
Stilbenes	0.5	0.0	4.0	1.00 (ref)	1.04 (0.90–1.21)	1.05 (0.90–1.23)	0.98 (0.79–1.21)	0.66	1.00 (0.97–1.03)
Lignans	1.4	0.8	2.9	1.00 (ref)	1.07 (0.92–1.25)	1.07 (0.91–1.26)	0.99 (0.81–1.20)	0.65	0.98 (0.90–1.08)
Other polyphenol classes									
Alkylphenols	25.0	2.9	65.3	1.00 (ref)	1.04 (0.87–1.25)	1.08 (0.88–1.31)	1.00 (0.81–1.24)	0.79	1.01 (0.96–1.05)
Tyrosols	3.2	0.5	16.1	1.00 (ref)	0.95 (0.82–1.10)	1.03 (0.87–1.23)	1.07 (0.83–1.37)	0.48	1.01 (0.97–1.04)
Alkylmethoxyphenols	2.3	0.3	5.0	1.00 (ref)	1.03 (0.88–1.21)	1.06 (0.90–1.24)	1.15 (0.97–1.35)	0.08	1.02 (0.99–1.06)
Methoxyphenols	0.3	0.0	0.7	1.00 (ref)	0.97 (0.83–1.14)	1.10 (0.95–1.28)	1.11 (0.94–1.30)	0.12	1.01 (0.98–1.04)
Hydroxybenzaldehydes	0.1	0.0	0.9	1.00 (ref)	0.86 (0.74–1.00)	0.90 (0.76–1.06)	0.90 (0.72–1.11)	0.76	0.98 (0.96–1.01)
Hydroxyphenylpropenes	0.0	0.0	2.2	1.00 (ref)	1.27 (0.99–1.63)	1.13 (0.92–1.39)	0.99 (0.79–1.26)	0.32	0.96 (0.93–1.00)
Other polyphenols	2.8	0.9	6.1	1.00 (ref)	0.99 (0.85–1.15)	1.06 (0.91–1.23)	1.14 (0.97–1.33)	0.06	1.02 (0.97–1.06)

Abbreviations: CI confidence interval, HR hazard ratio; P10 and P90 10th and 90th percentile. * *p*-value < 0.05; no association exceeds the Bonferroni threshold (*p* < 0.05/23) < 0.002. Model 2: stratified by study centre and age at baseline (1-year interval) and adjusted for body mass index, smoking status, alcohol consumption, education level, physical activity, menopausal status, age at menopause, age at first menstrual period, use of oral contraceptives, duration of oral contraceptives, hormone replacement therapy use, and full-term pregnancies.

**Table 3:** Hazard ratios and 95% confidence intervals for histological subtypes of epithelial ovarian cancer, according to quartile of intake of total polyphenols, flavonoids and phenolic acids in the EPIC study.

	Serous (Cases = 806)	Endometrioid (Cases = 129)	Mucinous (Cases = 102)	Clear Cell (Cases = 67)
	HR (95% CI)	HR (95% CI)	HR (95% CI)	HR (95% CI)
Total polyphenols				
Quartile 1	1.00 (ref)	1.00 (ref)	1.00 (ref)	1.00 (ref)
Quartile 2	1.03 (0.83–1.28)	0.77 (0.45–1.30)	1.10 (0.59–2.06)	0.76 (0.35–1.63)
Quartile 3	1.10 (0.87–1.41)	0.69 (0.38–1.24)	1.20 (0.61–2.37)	0.80 (0.34–1.86)
Quartile 4	1.16 (0.89–1.51)	0.78 (0.41–1.48)	1.47 (0.70–3.09)	1.04 (0.42–2.57)
*p*-trend	0.24	0.59	0.27	0.76
Continuous (log_2_)	1.12 (0.98–1.28)	1.10 (0.78–1.54)	1.27 (0.86–1.88)	1.07 (0.69–1.68)
Flavonoids				
Quartile 1	1.00 (ref)	1.00 (ref)	1.00 (ref)	1.00 (ref)
Quartile 2	1.03 (0.83–1.27)	1.13 (0.67–1.90)	1.50 (0.82–2.72)	1.41 (0.69–2.88)
Quartile 3	0.98 (0.77–1.24)	1.05 (0.59–1.88)	1.13 (0.57–2.26)	0.78 (0.31–1.92)
Quartile 4	1.13 (0.87–1.46)	0.88 (0.46–1.69)	1.09 (0.51–2.32)	1.27 (0.52–3.09)
*p*-trend	0.31	0.53	0.75	0.73
Continuous (log_2_)	1.04 (0.95–1.14)	0.98 (0.79–1.23)	0.96 (0.75–1.23)	1.12 (0.82–1.53)
Phenolic acids				
Quartile 1	1.00 (ref)	1.00 (ref)	1.00 (ref)	1.00 (ref)
Quartile 2	1.05 (0.85–1.30)	1.38 (0.82–2.32)	1.02 (0.54–1.92)	0.88 (0.43–1.80)
Quartile 3	1.14 (0.91–1.41)	0.87 (0.49–1.54)	1.39 (0.75–2.57)	1.01 (0.50–2.03)
Quartile 4	1.24 (0.99–1.56)	1.08 (0.60–1.93)	1.44 (0.75–2.76)	0.80 (0.35–1.80)
*p*-trend	0.04	0.83	0.19	0.66
Continuous (log_2_)	1.07 (0.98–1.16)	1.06 (0.85–1.30)	1.26 (0.98–1.63)	0.92 (0.70–1.21)

Abbreviations: CI confidence interval, HR hazard ratio. Model 2: stratified by study centre and age at baseline (1-year interval) and adjusted for body mass index, smoking status, alcohol consumption, education level, physical activity, menopausal status, age at menopause, age at first menstrual period, use of oral contraceptives, duration of oral contraceptives, hormone replacement therapy use, and full-term pregnancies.

## Data Availability

For information on how to submit an application for gaining access to EPIC data, please follow the instructions at http://epic.iarc.fr/access/index.php (Accessed on 1 June 2021).

## Supplementary Materials

The following are available online at https://www.mdpi.com/article/10.3390/antiox10081249/s1, Table S1: Baseline characteristics according to quartiles of total polyphenol intake in the EPIC study; Table S2: Hazard ratios and 95% confidence intervals for epithelial ovarian cancer, according to quartile of intake of total polyphenols, flavonoids and phenolic acids by coffee intake categories in the EPIC study; Table S3: Hazard ratios and 95% confidence intervals for epithelial ovarian cancer, according to quartile of intake of total polyphenols, flavonoids and phenolic acids by body mass index categories in the EPIC study; Figure S1: Map of the participating centres in the European Prospective Investigation into Cancer and Nutrition study.
