# Supplementary material for: Polyphenol Intake and Epithelial Ovarian Cancer Risk in the European Prospective Investigation into Cancer and Nutrition (EPIC) Study
Source: Antioxidants (Basel). 2021 Aug 4;10(8):1249. doi: 10.3390/antiox10081249 (PMC8389235; doi:10.3390/antiox10081249)
Supplement: Supplementary file 1 [file antioxidants-10-01249-s001.zip › antioxidants-1274069-supplementary.pdf]

**Supplementary Table S1.** Baseline characteristics according to quartiles of total polyphenol intake in the EPIC study.

| | Quartiles of total polyphenol intake | | | | | | | |
|---|---|---|---|---|---|---|---|---|
| | Quartile 1 | | Quartile 2 | | Quartile 3 | | Quartile 4 | |
| **Baseline characteristics** | **Mean** | **SD** | **Mean** | **SD** | **Mean** | **SD** | **Mean** | **SD** |
| N | 77,283 | | 77,282 | | 77,282 | | 77,282 | |
| Cut-off polyphenol intake (mg/d) | <910 | | 910–1,260 | | 1,261–1,918 | | >1,918 | |
| Age at recruitment (years) | 49.5 | 9.0 | 50.0 | 9.6 | 51.1 | 10.1 | 51.5 | 9.8 |
| BMI (kg/m2) | 25.3 | 4.6 | 24.8 | 4.3 | 24.6 | 4.2 | 24.4 | 4.1 |
| Alcohol intake (g/d) | 4.3 | 7.3 | 7.3 | 10.1 | 9.8 | 12.3 | 12.3 | 15.3 |
| Total energy intake (kcal/d) | 1,688 | 463 | 1,878 | 491 | 1,998 | 515 | 2,184 | 566 |
| Number of full term pregnancies | 2.0 | 1.3 | 1.9 | 1.2 | 1.9 | 1.2 | 1.9 | 1.2 |
| Age at first menstrual period (years) | 13.1 | 1.5 | 13.1 | 1.5 | 13.1 | 1.5 | 13.0 | 1.6 |
| Age at menopause (years) | 48.9 | 4.6 | 49.0 | 4.6 | 49.0 | 4.8 | 49.0 | 4.9 |
| Duration of oral contraceptive (years) | 6.5 | 6.8 | 7.9 | 7.5 | 8.5 | 7.5 | 8.2 | 7.4 |
| **Baseline characteristics** | **N** | **%** | **N** | **%** | **N** | **%** | **N** | **%** |
| Smoking status | | | | | | | | |
| Never | 44,454 | 57.5 | 41,717 | 54.0 | 42,380 | 54.8 | 40,744 | 54.8 |
| Former | 16,063 | 20.8 | 17,514 | 22.7 | 18,773 | 24.3 | 19,997 | 23.4 |
| Current | 14,922 | 19.3 | 16,645 | 21.5 | 14,565 | 18.9 | 14,446 | 19.6 |
| Not specified | 1,844 | 2.4 | 1,406 | 1.8 | 1,564 | 2.0 | 2,095 | 2.2 |
| Physical activity | | | | | | | | |
| Inactive | 19,125 | 24.8 | 15,843 | 20.5 | 13,827 | 17.9 | 13,174 | 17.1 |
| Moderately inactive | 24,698 | 32.0 | 26,925 | 34.8 | 27,307 | 35.3 | 26,920 | 34.8 |
| Moderately active | 23,366 | 30.2 | 21,580 | 27.9 | 20,521 | 26.6 | 21,227 | 27.5 |
| Active | 8,365 | 10.8 | 11,428 | 14.8 | 14,126 | 18.3 | 15,012 | 19.4 |
| Not specified | 1,729 | 2.2 | 1,506 | 2.0 | 1,501 | 1.9 | 949 | 1.2 |
| Educational level | | | | | | | | |
| None | 6,800 | 8.8 | 2,387 | 3.1 | 957 | 1.2 | 374 | 0.5 |
| Primary school completed | 21,978 | 28.4 | 20,348 | 26.3 | 15,528 | 20.1 | 12,438 | 16.1 |
| Technical/professional school | 16,422 | 21.3 | 17,655 | 22.8 | 17,968 | 23.3 | 17,139 | 22.2 |
| Secondary school | 17,823 | 23.1 | 18,289 | 23.7 | 19,175 | 24.8 | 19,361 | 25.1 |
| University | 13,067 | 16.9 | 16,649 | 21.5 | 19,705 | 25.5 | 22,591 | 29.2 |
| Not specified | 1,193 | 1.5 | 1,954 | 2.5 | 3,949 | 5.1 | 5,379 | 7.0 |
| Menopausal status | | | | | | | | |
| Premenopausal | 29,916 | 38.7 | 29,074 | 37.6 | 26,584 | 34.4 | 25,013 | 32.4 |
| Postmenopausal | 30,657 | 39.7 | 32,314 | 41.8 | 36,164 | 46.8 | 36,860 | 47.7 |
| Perimenopausal | 16,710 | 21.6 | 15,894 | 20.6 | 14,534 | 18.8 | 15,409 | 19.9 |
| Ever had any live born children | | | | | | | | |
| No | 3,727 | 4.8 | 2,618 | 3.4 | 1,962 | 2.5 | 1,850 | 2.4 |
| Yes | 61,159 | 79.1 | 57,561 | 74.5 | 57,734 | 74.7 | 60,473 | 78.3 |
| Not specified | 12,397 | 16.0 | 17,103 | 22.1 | 17,586 | 22.8 | 14,959 | 19.4 |
| Full term pregnancy | | | | | | | | |
| No | 10,035 | 13.0 | 10,910 | 14.1 | 12,184 | 15.8 | 12,278 | 15.9 |
| Yes | 64,035 | 82.9 | 62,299 | 80.6 | 61,739 | 79.9 | 61,907 | 80.1 |
| Not specified | 3,213 | 4.2 | 4,073 | 5.3 | 3,359 | 4.4 | 3,097 | 4.0 |
| Ever use of oral contraceptive | | | | | | | | |
| No | 33,443 | 43.3 | 28,883 | 37.4 | 26,723 | 34.6 | 26,284 | 34.0 |
| Yes | 41,445 | 53.6 | 45,426 | 58.8 | 48,671 | 63.0 | 49,894 | 64.6 |
| Not specified | 2,395 | 3.1 | 2,973 | 3.9 | 1,888 | 2.4 | 1,104 | 1.4 |

| | | | | | | | | |
|---|---|---|---|---|---|---|---|---|
| Duration of oral contraceptive (years) | | | | | | | | |
| ≤1 | 9,950 | 12.9 | 8,577 | 11.1 | 7,431 | 9.6 | 7,278 | 9.4 |
| 2 – 4 | 10,728 | 13.9 | 9,693 | 12.5 | 9,352 | 12.1 | 10,136 | 13.1 |
| 5 – 7 | 6,388 | 8.3 | 6,780 | 8.8 | 7,838 | 10.1 | 7,873 | 10.2 |
| 8 – 10 | 4,934 | 6.4 | 6,214 | 8.0 | 7,407 | 9.6 | 7,370 | 9.5 |
| >10 | 7,096 | 9.2 | 10,367 | 13.4 | 11,737 | 15.2 | 10,680 | 13.8 |
| Not specified | 38,187 | 49.4 | 35,651 | 46.1 | 33,517 | 43.4 | 33,945 | 43.9 |
| Ever use of hormone replacement therapy | | | | | | | | |
| No | 55,025 | 71.2 | 52,652 | 68.1 | 52,713 | 68.2 | 52,781 | 68.3 |
| Yes | 16,394 | 21.2 | 17,352 | 22.5 | 19,344 | 25.0 | 21,438 | 27.7 |
| Not specified | 5,864 | 7.6 | 7,278 | 9.4 | 5,225 | 6.8 | 3,063 | 4.0 |
| Ever breast-feed | | | | | | | | |
| No | 16,702 | 21.6 | 18,033 | 23.3 | 20,773 | 26.9 | 21,908 | 28.4 |
| Yes | 52,881 | 68.4 | 48,365 | 62.6 | 46,985 | 60.8 | 49,115 | 63.6 |
| Not specified | 7,700 | 10.0 | 10,884 | 14.1 | 9,524 | 12.3 | 6,259 | 8.1 |

**Supplementary Table S2.** Hazard ratios and 95% confidence intervals for epithelial ovarian cancer, according to quartile of intake of total polyphenols, flavonoids and phenolic acids by coffee intake categories in the EPIC study.

| | Coffee non-consumers | Coffee consumers |
|---|---|---|
| | **HR (95% CI)** | **HR (95% CI)** |
| N | 24,582 | 284,302 |
| Cases | 96 | 1,371 |
| **Total polyphenols** | | |
| Quartile 1 | 1.00 (ref) | 1.00 (ref) |
| Quartile 2 | 0.65 (0.33-1.28) | 1.05 (0.89-1.24) |
| Quartile 3 | 0.67 (0.32-1.40) | 1.10 (0.92-1.33) |
| Quartile 4 | 0.41 (0.17-0.94) | 1.23 (1.00-1.50) |
| P-trend | 0.04 | 0.05 |
| Continuous (log2) | 0.87 (0.63-1.21) | 1.17 (1.04-1.30) |
| **Flavonoids** | | |
| Quartile 1 | 1.00 (ref) | 1.00 (ref) |
| Quartile 2 | 0.57 (0.29-1.1) | 1.03 (0.88-1.22) |
| Quartile 3 | 0.61 (0.30-1.24) | 1.06 (0.88-1.27) |
| Quartile 4 | 0.50 (0.23-1.09) | 1.11 (0.91-1.36) |
| P-trend | 0.30 | 0.12 |
| Continuous (log2) | 0.89 (0.70-1.15) | 1.04 (0.97-1.12) |
| **Phenolic acids** | | |
| Quartile 1 | 1.00 (ref) | 1.00 (ref) |
| Quartile 2 | 0.65 (0.32-1.29) | 0.98 (0.83-1.15) |
| Quartile 3 | 0.65 (0.30-1.40) | 1.04 (0.88-1.22) |
| Quartile 4 | 0.55 (0.23-1.28) | 1.16 (0.97-1.38) |
| P-trend | 0.08 | 0.23 |
| Continuous (log2) | 0.80 (0.56-1.14) | 1.11 (1.03-1.19) |
| **Hydroxycinnamic acids** | | |
| Quartile 1 | 1.00 (ref) | 1.00 (ref) |

| | | |
|---|---|---|
| Quartile 2 | 1.05 (0.56-1.95) | 1.11 (0.95-1.3) |
| Quartile 3 | 0.59 (0.27-1.27) | 1.06 (0.9-1.25) |
| Quartile 4 | 0.80 (0.35-1.79) | 1.17 (0.99-1.39) |
| P-trend | 0.12 | 0.33 |
| Continuous (log2) | 0.78 (0.52-1.16) | 1.08 (1.01-1.15) |

Abbreviations: BMI body mass index; CI confidence interval, HR hazard ratio

Model 2: stratified by study centre and age at baseline (1-year interval) and adjusted for BMI, smoking status, alcohol consumption, education level, physical activity, menopausal status, age at menopause, age at first menstrual period, use of oral contraceptives, duration of oral contraceptives, hormone replacement therapy use, and full-term pregnancies.

**Supplementary Table S3.** Hazard ratios and 95% confidence intervals for epithelial ovarian cancer, according to quartile of intake of total polyphenols, flavonoids and phenolic acids by body mass index categories in the EPIC study.

| | BMI < 25 | BMI 25 – 29.9 | BMI ≥ 30 | P for interaction |
|---|---|---|---|---|
| | HR (95% CI) | HR (95% CI) | HR (95% CI) | |
| N | 186,764 | 87,590 | 34,775 | |
| Cases | 808 | 451 | 210 | |
| Total polyphenols | | | | 0.018 |
| Quartile 1 | 1.00 (ref) | 1.00 (ref) | 1.00 (ref) | |
| Quartile 2 | 0.99 (0.80-1.22) | 1.18 (0.87-1.60) | 1.08 (0.67-1.76) | |
| Quartile 3 | 0.96 (0.75-1.22) | 1.22 (0.88-1.71) | 1.58 (0.96-2.59) | |
| Quartile 4 | 1.05 (0.81-1.36) | 1.41 (0.98-2.03) | 1.61 (0.93-2.82) | |
| P-trend | 0.89 | 0.07 | 0.15 | |
| Continuous ($log_2$) | 1.06 (0.92-1.21) | 1.18 (0.98-1.42) | 1.23 (0.95-1.60) | |
| Flavonoids | | | | 0.25 |
| Quartile 1 | 1.00 (ref) | 1.00 (ref) | 1.00 (ref) | |
| Quartile 2 | 0.90 (0.72-1.11) | 1.13 (0.84-1.53) | 1.01 (0.66-1.53) | |
| Quartile 3 | 0.93 (0.73-1.17) | 1.34 (0.97-1.85) | 1.10 (0.70-1.72) | |
| Quartile 4 | 0.99 (0.77-1.28) | 1.37 (0.96-1.95) | 0.99 (0.77-1.28) | |
| P-trend | 0.73 | 0.11 | 0.47 | |
| Continuous ($log_2$) | 1.01 (0.92-1.11) | 1.07 (0.94-1.21) | 0.98 (0.83-1.17) | |
| Phenolic acids | | | | 0.09 |
| Quartile 1 | 1.00 (ref) | 1.00 (ref) | 1.00 (ref) | |
| Quartile 2 | 1.09 (0.88-1.34) | 0.95 (0.71-1.25) | 0.98 (0.63-1.55) | |
| Quartile 3 | 1.18 (0.96-1.46) | 1.01 (0.76-1.35) | 0.97 (0.61-1.55) | |
| Quartile 4 | 1.09 (0.86-1.37) | 1.24 (0.91-1.67) | 1.57 (0.99-2.51) | |
| P-trend | 0.50 | 0.09 | 0.02 | |
| Continuous ($log_2$) | 1.04 (0.96-1.13) | 1.07 (0.95-1.21) | 1.19 (1.01-1.41) | |

Abbreviations: BMI body mass index; CI confidence interval, HR hazard ratio

Model 2: stratified by study centre and age at baseline (1-year interval) and adjusted for BMI, smoking status, alcohol consumption, education level, physical activity, menopausal status, age at menopause, age at first menstrual period, use of oral contraceptives, duration of oral contraceptives, hormone replacement therapy use, and full-term pregnancies.

**Supplementary Figure S1**

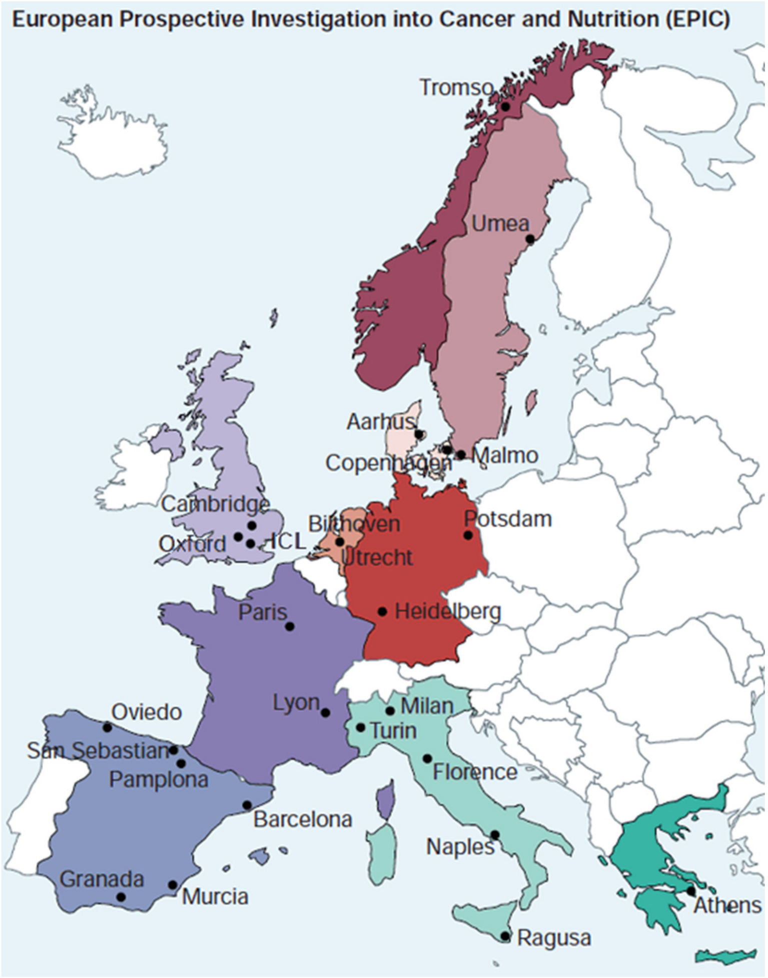


**Supplementary Figure S1.** Map of the participating centres in the European Prospective Investigation into Cancer and Nutrition study. All centres of France, Utrecht (The Netherlands) and Florence only recruited women. Data from Greece was not available for the present study. ICL: Imperial College of London. Reproduced from https://epic.iarc.fr/centers/epicmap.php. Accessed on 2021-07-22.
